# From Aquaculture to Aquaculture: Production of the Fish Feed Additive Astaxanthin by *Corynebacterium glutamicum* Using Aquaculture Sidestream

**DOI:** 10.3390/molecules28041996

**Published:** 2023-02-20

**Authors:** Ina Schmitt, Florian Meyer, Irene Krahn, Nadja A. Henke, Petra Peters-Wendisch, Volker F. Wendisch

**Affiliations:** Institute for Genetics of Prokaryotes, Faculty of Biology and CeBiTec, Bielefeld University, 33615 Bielefeld, Germany

**Keywords:** *Corynebacterium glutamicum*, astaxanthin, RAS, aquaculture sidestream, circular economy

## Abstract

Circular economy holds great potential to minimize the use of finite resources, and reduce waste formation by the creation of closed-loop systems. This also pertains to the utilization of sidestreams in large-scale biotechnological processes. A flexible feedstock concept has been established for the industrially relevant *Corynebacterium glutamicum*, which naturally synthesizes the yellow C50 carotenoid decaprenoxanthin. In this study, we aimed to use a preprocessed aquaculture sidestream for production of carotenoids, including the fish feed ingredient astaxanthin by *C. glutamicum*. The addition of a preprocessed aquaculture sidestream to the culture medium did not inhibit growth, obviated the need for addition of several components of the mineral salt’s medium, and notably enhanced production of astaxanthin by an engineered *C. glutamicum* producer strain. Improved astaxanthin production was scaled to 2 L bioreactor fermentations. This strategy to improve astaxanthin production was shown to be transferable to production of several native and non-native carotenoids. Thus, this study provides a proof-of-principle for improving carotenoid production by *C. glutamicum* upon supplementation of a preprocessed aquaculture sidestream. Moreover, in the case of astaxanthin production it may be a potential component of a circular economy in aquaculture.

## 1. Introduction

The consumer demand for seafood is rising as the world population is growing and healthier diets are becoming more important. The worldwide fish production was 178.5 million tons (mt) in 2018, and is anticipated to reach 204.4 mt in 2030 [1]. This tremendous demand for fish cannot be met by fishery alone. Accordingly, aquaculture is the fastest-growing food production system [2], enabling the fishing industry to meet the global needs [3,4]. The amount of seafood bred in aquaculture systems contributed 46% of the global production (82.1 mt) in 2018, and this share is expected to reach 53% (108.5 mt) in 2030 [1].

Recirculating aquaculture systems (RAS) present a promising alternative to the traditional cultivation systems as they have greatly reduced land and water requirements, and can be built without exploitation of farmland [5,6]. Moreover, they offer year-round fish growth and provide a high degree of environmental control [7,8]. RAS have great opportunities for waste management and nutrient recycling [9]. The lower flow rates of RAS compared to raceway systems, and the high stocking densities of RAS compared to ponds and cages lead to lesser, more concentrated effluents from the fish tanks, which can be treated more cost effectively [2,7,9,10]. RAS and other aquaculture effluents are mainly composed of settleable and dissolved nutrients from feces and unconsumed fish feed [11], which need to be removed in order to reuse the water for the fish tanks. Via settling of the backwash water, a sludge phase containing most of the settleable wastes and an aqueous sidestream can be obtained [7,12]. Several efforts have been made to recycle this solid and aqueous sidestreams from RAS. For instance, the aqueous phase of the waste streams has been used for the cultivation of several algae [13,14,15]. The algal biomass may be used as natural fertilizer [15,16], an aquaculture feed [17,18], or food [19] ingredient. Alternatively, the algae can be used for the extraction of high value compounds, such as polyunsaturated fatty acids [20] or vitamins [21]. Further technological developments are in demand to utilize these aquaculture sidestreams.

One of the most important quality criteria of aquaculture bred fish, such as salmon (2.4 mt in 2018) and rainbow trout (0.8 mt in 2018), and crustaceans (9.4 mt in 2018) [1], is the degree of flesh and/or shell pigmentation, as it dictates their market value [22,23]. As fish and other aquatic animals are unable to synthesize the responsible carotenoids *de novo* carotenoids are mixed into the feed of farmed fish and crustaceans [24,25]. Astaxanthin and other carotenoids from synthetic [23,26,27] and from natural sources, such as yeasts [28,29], algae [30,31,32], maize [33], and bacteria [22,34], have been used in aquatic feed formulations, achieving different levels of coloration. The red, cyclic C40 carotenoid astaxanthin is the major carotenoid used in aquatic feeds [25]. The global astaxanthin market is predicted to reach 4.75 billion US$ in 2028, with an annual growth rate of 16.8% [35]. Currently, the petrochemical synthesis of astaxanthin is the most cost-efficient and, therefore, dominates the market [24,36]. However, there is a growing trend towards naturally sourced carotenoids due to consumer demand and regulation. This opens the market for alternative production technologies. Furthermore, it was shown that the antioxidant properties of natural astaxanthin from *Haematococcus pluvialis* are stronger than those of synthetic astaxanthin [37]. However, studies with rainbow trout showed that the esterified astaxanthin produced by microalgae are deposited in fish muscle less effectively than the free variant, and therefore cause lower coloration [38,39]. Taken together, the demand for natural, unesterified astaxanthin that can be produced efficiently and environmentally friendly is rising. Microbial hosts, such as *Yarrowia lipolytica* [40,41], *Paracoccus carotinifaciens* [42], *Escherichia coli* [43,44], *Saccharomyces cerevisiae* [45], and *Corynebacterium glutamicum* [46] are natural or heterologous producers of carotenoids and have been engineered for high-level production of astaxanthin.

**Figure 1:**
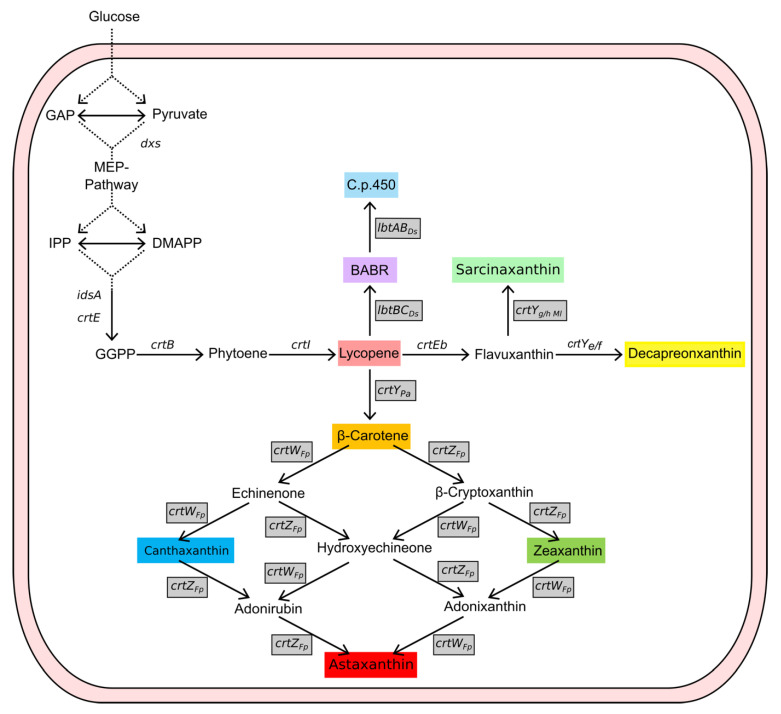
**Carotenoid Biosynthesis in *C. glutamicum*.** Gene names are given next to the reactions catalyzed by their gene products. Heterologous genes are depicted with a grey box. GAP: Glyceraldehyde 3-phosphate; IPP: isopenthenyl pyrophosphate; DMAPP: dimethylallyl diphosphate; BABR: bisanhydrobacterioruberin; C.p.450: 2,2′-bis-(4-hydroxy-3-methybut-2enyl)-β,β-carotene; *dxs*: 1-deoxy-D-xylulose 5-phosphate synthase; *idsA*: geranylgeranyl pyrophosphate synthase; *crtE*: geranylgeranyl pyrophosphate synthase; *crtB*: phytoene synthase, *crtI*: phytoene desaturase; *crtEb*: lycopene elongase; *crtY_e_*_/*f*_: ϵ-cyclase; *crtY_g_*_/*h Ml*_: C50 carotenoid γ-cyclase from *Micrococcus leuteus*; *lbtBC_Ds_*: subunit of C50 carotenoid β-cyclase (B) and lycopene elongase (C) from *Dietzia* sp. CQ4; *lbtAB_Ds_*: C50 carotenoid β-cyclase from *Dietzia* sp. CQ4; *crtY_Pa_*: lycopene cyclase from *Pantoea ananatis*; *crtW_Fp_*: β-carotene ketolase from *Fulvimarina pelagi*; *crtZ_Fp_*: β-carotene hydroxylase from *Fulvimarina pelagi*.

Astaxanthin can be synthesized by the Gram^+^ soil bacterium *C. glutamicum* that is a natural producer of the yellow C50 carotenoid decaprenoxanthin and its glucosides [47] (Figure 1). Metabolic engineering is effective for *C. glutamicum*, which efficiently produces the endogenous decaprenoxanthin [48] and lycopene [49], and the heterologous isoprenoids α-pinene [50], (+)-valencene [51], 4-apolycopene and 4-aponeurosporene [52], patchoulol [53], α-farnesene [54] α-carotene [55], bisanhydrobacterioruberin (BABR) [56], C.p.450 and sarcinaxanthin [57], β-carotene [57], and astaxanthin [46]. In order to achieve high level astaxanthin production several engineering strategies have been applied. First, the carotenoid biosynthesis pathway was terminated at lycopene by deletion of *crtY_e_Y_f_Eb* [49,58], and the precursor supply was improved [58,59]. Furthermore, the regulatory mechanism of the carotenoid biosynthesis by its precursor molecule geranylgeranyl pyrophosphate (GGPP) responsive transcriptional repressor CrtR was elucidated [57,60] and deletion of its gene was shown to improve astaxanthin production [46]. Conversion of β-carotene to astaxanthin was improved by a fusion protein of the heterologous β-carotene hydroxylase and β-carotene ketolase from *Fulvimarina pelagi* [46]. A CRISPRi library screening identified potential further targets for metabolic engineering [61].

In addition to genetic engineering, other attempts have been made to optimize the carotenoid production by microbial hosts and to design sustainable production processes. Successful strategies include: two-stage cultivations [62,63], the adjustment of operative bioprocess parameters, such as aeration rate [64], CO_2_ levels [65], light irradiation [66,67], temperature [68,69], pH [70,71], optimization of the growth medium composition [72,73], and utilization of various sustainable carbon sources [64,74,75,76].

*C. glutamicum* has been engineered for utilization of agricultural sidestreams [77]. This is relevant since the bacterium is used for decades in the production of amino acids at the million ton scale [78]. The flexible feedstock concept for *C. glutamicum* comprises efficient utilization for growth and production of a number of compounds, for instance, the lignocellulosic sugars arabinose [79,80] and xylose [81,82,83,84], the chitin derived amino sugars glucosamine [85] and *N*-acetyl-glucosamine [86]. Furthermore, also less processed substrates, such as chitin [87], and hydrolysates of plant biomass, such as rice straw or wheat bran [88,89,90], and sidestreams from biodiesel factories [91], biorefineries [92], or the starch and paper industry [93,94] were harnessed as carbon sources for *C. glutamicum*.

Here, we studied if a sidestream from a Norwegian salmon farm, operated as a RAS, can be utilized as component of the growth medium to support growth and carotenoid production by *C. glutamicum*.

## 2. Results

### 2.1. Analysis of the Untreated Aquaculture Sidestream

**Figure 2:**
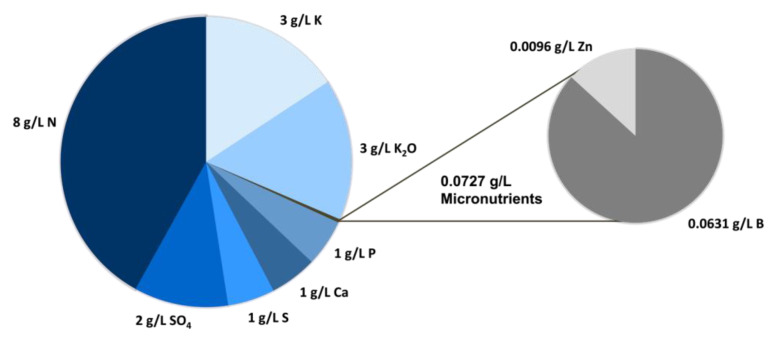
**Nutrient composition of the aquaculture sidestream.** Macro- and Micro-nutrients detected in an analysis of the untreated aquaculture sidestream performed by Eurofins Agraranalytik Deutschland GmbH. Nutrients below the detection limit are not represented.

The liquid phase of an aquaculture sidestream from a Norwegian RAS salmon farm was analyzed regarding its physical parameters and nutrient composition in order to get insights about which components of the standard minimal growth medium CGXII of *C. glutamicum* might be substitutable by the aquaculture sidestream. The aquaculture sidestream has a pH of 5.5, and a dry matter fraction of 16.5 g L^−1^. In a loss on ignition analysis an organic fraction could not be detected (detection limit 0.1% (*w*/*w*) of dry matter fraction). The nutrient analysis of the aquaculture sidestream (Figure 2) showed that nitrogen (8 g L^−1^) is the major macronutrient present in the aquaculture sidestream, followed by potassium (3 g L^−1^ K, 3 g L^−1^ K_2_O) and sulphate (2 g L^−1^). Furthermore, elemental sulfur, phosphorus, calcium, zinc, and boron were detected. The analysis also covered ammonium derived nitrogen (NH_4_-N) (<1 g L^−1^), P_2_O_5_ (<3 g L^−1^), CaO (<2 g L^−1^), Mg (<1 g L^−1^), MgO (<2 g L^−1^), Mn, Cu, and Mo (all <0.0000485 g L^−1^), but these nutrients could not be detected.

Furthermore, the amino acid and amine composition of preprocessed aquaculture sidestream (AQ) was analyzed via HPLC. The results (Table S1) display that besides proteinogenic amino acids, the non-proteinogenic *ω*-amino acid 5-aminovaleric acid (5-AVA), and the diamines putrescine and cadaverine are present in AQ. The concentrations of the proteinogenic amino acids range from 0.5 to 10.7 mg L^−1^, while 5-AVA (76 mg L^−1^), putrescine (62 mg L^−1^), and cadaverine (53 mg L^−1^) are present in higher concentrations.

Moreover, an ion exchange chromatography (detection with a RID at λ = 210 nm) analysis of AQ was performed via HPLC (Figure S2). Four Peaks were detected. However, none of the peaks could be identified. Glucose, malate, lactate, trehalose, succinate, and α-ketoglutarate were used as standards and can therefore be excluded as possible carbohydrate components of the AQ.

### 2.2. Growth in Various Media Based on or Supplemented with AQ

**Figure 3:**
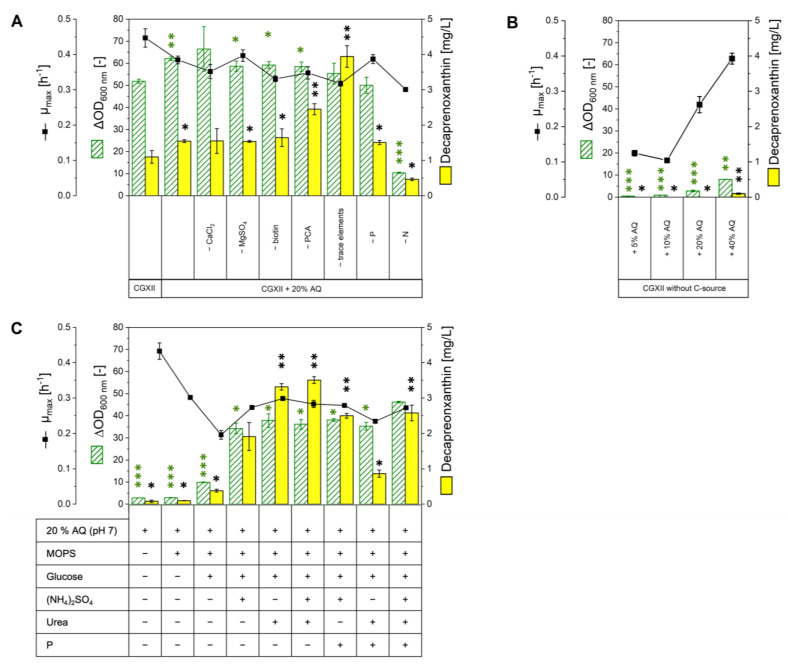
**Growth of *C. glutamicum* WT in AQ containing media.** ∆OD_600 nm_, µ_max,_ and decaprenoxanthin production after 48 h of *C. glutamicum* WT grown in a Biolector^®^ flowerplate microcultivation system. The maximal OD_600 nm_ difference from the initial OD_600 nm_ during 48 h of cultivation is given as ∆OD_600 nm_. Values and error bars represent means and standard deviations of triplicate cultivations. Statistical significance in comparison to the cultivation in CGXII medium was assessed for ∆OD_600 nm_ (marked in green) and decaprenoxanthin production (marked in black) in Student’s *t*-test (*** *p* < 0.0001, ** *p* < 0.001, * *p* < 0.05). (**A**) Growth on CGXII, CGXII with addition of 20% (*v*/*v*) AQ and CGXII with 20% (*v*/*v*) AQ replacing media components of the CGXII composition. (**B**) Growth on CGXII without carbon source, 5 to 40% (*v*/*v*) AQ were supplemented as replacement. (**C**) Growth on AQ as the sole medium component, with adjustment to pH 7 and the addition of MOPS buffer, glucose, (NH_4_)_2_SO_4_, and/or urea and phosphorous source (P). The last column represents the AQ based medium CGAQ.

In order to use the aquaculture sidestream as a growth medium component, it was preprocessed. Centrifugation and subsequent sterile filtration (Section 4.1) were applied to obtain a clear liquid. *C. glutamicum* WT was used to verify if AQ as a new complex media component is compatible with growth. Therefore, cultivations in different media compositions were performed in a Biolector^®^ microcultivation system. Addition of AQ to the standard minimal growth medium CGXII to 20% (*v*/*v*) led to significantly increased biomass formation (from OD_600 nm_ 52.0 ± 0.9 to 62.0 ± 0.8) and decaprenoxanthin content (from 1.1 ± 0.2 to 1.6 ± 0.0 mg L^−1^), while the growth rate was only slightly reduced (Figure 3A). To elucidate which components of CGXII could be replaced by the complex media component AQ, each component of the CGXII media composition was substituted by 20% (*v*/*v*) AQ (Figure 3A). Replacement of the CGXII components CaCl_2_, MgSO_4_, biotin, protocatechuic acid (PCA), or trace elements by AQ led to comparable biomass formation and growth rates compared to CGXII with addition of 20% (*v*/*v*) AQ. This indicated that addition of 20% (*v*/*v*) AQ is sufficient to replace these components of CGXII. However, replacing PCA or trace elements with 20% (*v*/*v*) AQ significantly increased decaprenoxanthin production (plus 58% for PCA; plus 155% for trace elements). The addition of AQ instead of phosphorous reduced biomass formation (∆OD_600 nm_ of 50 ± 3.7; minus 19%), but decaprenoxanthin production was comparable. The replacement of nitrogen had the strongest negative effect: biomass formation was reduced to one-fifth (∆OD_600 nm_ of 10 ± 0.3), the maximal growth rate by one-third (0.3 ± 0.0 h^−1^), and the decaprenoxanthin production by more than half (0.5 ± 0.0 mg L^−1^) compared to CGXII. To test whether AQ could function as a carbon source for *C. glutamicum*, the standard carbon source glucose was replaced by 5%, 10%, 20%, or 40% (*v*/*v*) AQ (Figure 3B). In all cases growth was observed, but even with the addition of 40% (*v*/*v*) AQ only one-sixth of biomass formation (∆OD_600 nm_ of 8.0 ± 0.0) was observed (Figure 3B). These results indicate that AQ can only partially substitute for the carbon and nitrogen sources of CGXII medium.

Second, we tested if *C. glutamicum* WT could grow with 20% (*v*/*v*) AQ (adjusted to pH 7) as sole medium component. However, the biomass and decaprenoxanthin formation were negligible (Figure 3C). When MOPS buffer (42 g L^−1^, adjusted to pH 7) and glucose were added, some growth (∆OD_600 nm_ of 10 ± 0.2) and decaprenoxanthin production (0.4 ± 0.0 mg L^−1^) were detected (Figure 3C). The addition of the nitrogen sources ammonium sulfate and/or urea increased biomass formation to ∆OD_600 nm_ of 34 ± 2.3, 38 ± 0.5 and 36 ± 2.1 for ammonium sulfate, urea, and both, respectively. Regarding decaprenoxanthin production, a significant increase was observed upon addition of urea alone (3.3 ± 0.1 mg L^−1^), or combined with ammonium sulfate (3.5 ± 0.1 mg L^−1^). The decaprenoxanthin titer was 3-fold higher than from the standard CGXII medium.

Third, we developed an AQ based growth medium for carotenoid production with *C. glutamicum*, in which all components that could be replaced by AQ without reducing biomass formation or decaprenoxanthin production (CaCl_2_, MgSO_4_, biotin, trace elements, and PCA) were omitted. The new medium was named CGAQ, and it contained 20% (*v*/*v*) AQ, 42 g L^−1^ MOPS buffer, 40 g L^−1^ glucose, 20 g L^−1^ (NH_4_)_2_SO_4_, 5 g L^−1^ urea, 1 g L^−1^ K_2_HPO_4_, and 1 g L^−1^ KH_2_PO_4_). Growth of *C. glutamicum* was supported by the medium CGAQ (Figure 3C). The biomass formation in CGAQ was comparable to the culture grown in CGXII, even though the growth rate dropped from 0.45 ± 0.02 h^−1^ to 0.27 ± 0.01 h^−1^. Notably, a more than doubled decaprenoxanthin content of 2.6 ± 0.2 mg L^−1^ was observed using medium CGAQ.

Taken together, we have developed two growth media using AQ that are suitable for *C. glutamicum*: CGAQ and the regular CGXII minimal medium supplemented with 20% (*v*/*v*) AQ.

### 2.3. Carotenoid Production in AQ Supplemented Media

**Figure 4:**
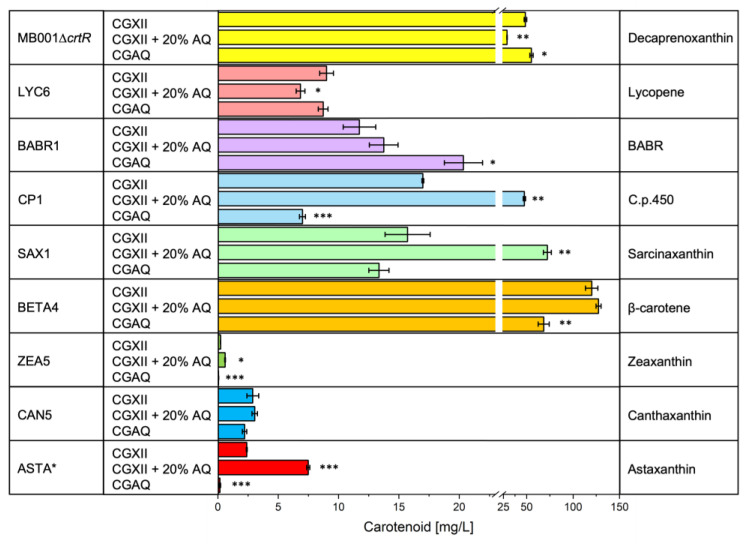
**Carotenoid production of *C. glutamicum* strains in AQ supplemented media.** Carotenoid production of *C. glutamicum* MB001∆*crtR* (decaprenoxanthin), LYC6 (lycopene), CP1 (C.p.450), BABR1 (bisanhydrobacterioruberin), SAX1 (sarcinaxanthin), BETA4 (β-carotene), ZEA5 (zeaxanthin), CAN5 (canthaxanthin), ASTA* (astaxanthin) grown on CGXII, CGXII supplemented with 20% (*v*/*v*) AQ, or the AQ derived medium CGAQ for 48 h. Values and error bars represent means and standard deviations of triplicate cultivations. Statistical significance in comparison to the cultivation of each strain in CGXII medium was assessed in Student´s *t*-test (*** *p* < 0.0001, ** *p* < 0.001, * *p* < 0.05).

As the new AQ based medium CGAQ more than doubled the decaprenoxanthin production, we further tested its impact on the production of other carotenoids. Therefore, we performed production experiments in the Biolector^®^ microcultivation system using strains overproducing various carotenoids. Strain ASTA* produces astaxanthin while ASTALYS* (strain construction see Section 4.4) is producing astaxanthin along with L-lysine. Strains LYC6, BETA4, ZEA5, and CAN5 (strain construction of ZEA5 and CAN5 see Section 4.4) produce the astaxanthin precursors lycopene, β-carotene, zeaxanthin, and canthaxanthin, respectively. Furthermore, we chose strains MB001∆*crtR*, BABR1, CP1, and SAX1 for production of the C50 carotenoids decaprenoxanthin, BABR, C.p.450, and sarcinaxanthin, respectively. All strains were grown either in CGXII, CGXII supplemented with 20% (*v*/*v*) AQ or CGAQ (Figure 4, Figure S3 and Figure S4).

Decaprenoxanthin and biomass formation by MB001∆*crtR* were increased in CGAQ (55.1 ± 1.8 mg L^−1^ decaprenoxanthin; plus 13%), but reduced by supplementation of CGXII with 20% (*v*/*v*) AQ (Figure 4). Lycopene formation by LYC6 in CGAQ (8.7 ± 0.4 mg L^−1^) was comparable to the one in CGXII, but was reduced by 24% when AQ was supplemented.

Production of BABR by strain BARBR1 was increased by supplementation of CGXII with 20% (*v*/*v*) AQ (13.7 ± 1.2 mg L^−1^; plus 17%), and further improved in CGAQ (20.3 ± 1.6 mg L^−1^; plus 73%), while the biomass formation was decreased in CGAQ. Production of two other non-native C50 carotenoids C.p.450 and sarcinaxanthin by strains CP1 and SAX1, respectively, was reduced in CGAQ as was biomass formation. However, upon addition of AQ to CGXII, carotenoid production increased considerably: plus 280% C.p.450 (47.5 ± 1.2 mg L^−1^), and plus 360% sarcinaxanthin (72.3 ± 4.3 mg L^−1^).

A similar pattern was observed for production of astaxanthin and its precursors by BETA4, ZEA5, CAN5, and ASTA*, respectively. In CGAQ, these strains exhibited reduced biomass and carotenoid formation. By contrast, the addition of AQ to CGXII had a positive impact on biomass formation and carotenoid production. While the slight increases in production of β-carotene (127.4 ± 2.7 mg L^−1^; plus 6%) and canthaxanthin (3.0 ± 0.2 mg L^−1^; plus 5%) were not significant compared to CGXII, considerable and statistically significant increases were observed for zeaxanthin (0.6 ± 0.0 mg L^−1^; plus 173%) and astaxanthin (7.4 ± 0.1 mg L^−1^; plus 213%).

The strain that co-produces astaxanthin with lysine, ASTALYS*, showed very low biomass formation compared to the other carotenoid production strains, which increased significantly upon addition of AQ to the medium (5.6 ± 0.2; plus 393%) or in the CGAQ medium (4.0 ± 0.2; plus 246%). Strain ASTALYS* showed 1.5-fold increased astaxanthin production (0.3 ± 0.0 mg L^−1^) upon addition of AQ to CGXII. Lysine production was increased 1.8-fold in CGAQ (1.40 ± 0.03 g L^−1^), and more than 6-fold (5.52 ± 0.14 g L^−1^) upon supplementation of CGXII with AQ.

For all strains, changes in the total carotenoid content (Figure S3) were observed. In case of strains MB001∆*crtR*, LYC6, and BETA4, all precursors were converted to decaprenoxanthin, lycopene and β-carotene, respectively. Here, changes in total carotenoids arose solely from changes in the respective product. In the other strains conversion of the precursors e.g., lycopene and β-carotene was incomplete, and therefore changes in the total carotenoid contents result from changes in product and precursor contents.

Taken together, the usage of AQ as a media component showed a positive effect on the carotenoid production by *C. glutamicum*. Notably, while production of the native carotenoid decaprenoxanthin was increased in CGAQ, supplementation of 20% AQ to CGXII improved production of most other carotenoids, in particular astaxanthin.

### 2.4. Fermentative Production of Astaxanthin in AQ Supplemented Media

**Figure 5:**
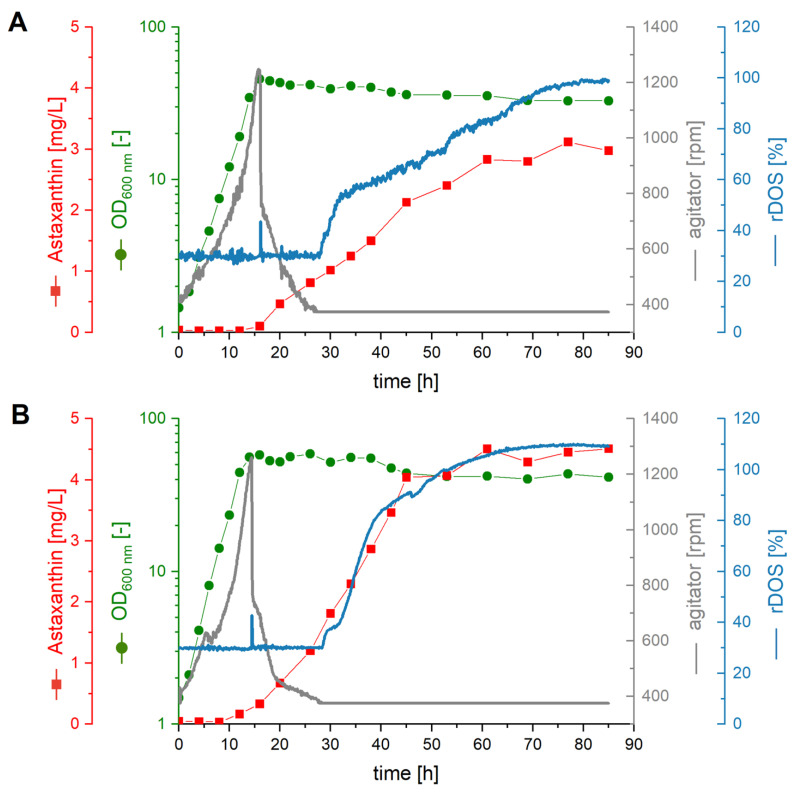
**Batch-Fermentations.** Progression of astaxanthin content (red squares), OD_600 nm_ (green dots), agitator speed (grey line), and relative dissolved oxygen concentration (rDOS) (blue line) over time during 2 L batch fermentations with *C. glutamicum* ASTA* grown in CGXII medium (**A**) and CGXII medium supplemented with 20% (*v*/*v*) AQ (**B**).

After having shown that astaxanthin production by *C. glutamicum* ASTA* was enhanced by the addition of 20% (*v*/*v*) AQ to CGXII medium in microcultivation, lab-scale bioreactors (2 L) were used to test if this improvement was stable at larger scale. Two parallel 2 L batch fermentations using *C. glutamicum* ASTA* were performed. They contained either CGXII medium (Figure 5A) or CGXII plus 20% (*v*/*v*) AQ (Figure 5B). After 16 h, both cultures reached the stationary phase. The biomass formation was higher upon AQ supplementation (∆OD_600 nm_ of 58 as compared to 46), as was the growth rate (0.31 h^−1^ compared 0.24 h^−1^). In CGXII, astaxanthin accumulated to a cellular content of 0.38 mg g^−1^ cell dry weight (CDW) (equivalent to 3.12 mg L^−1^) during 77 h, with a maximal volumetric productivity of 0.05 mg L^−1^ h^−1^ (Figure 5A). Upon AQ supplementation, the maximum astaxanthin concentration of 4.51 mg L^−1^ (cellular content of 0.44 mg g^−1^ CDW) was reached already after 61 h, increasing the maximal volumetric productivity to 0.09 mg L^−1^ h^−1^ (Figure 5B). Thus, astaxanthin production was considerably improved regarding titers and volumetric productivities in bioreactor batch cultivation indicating that the beneficial effect of AQ supplementation is transferable to larger scales under defined bioreactor conditions.

With the aim to improve astaxanthin production in bioreactor cultivation, fed-batch fermentations were performed. In a comparison, 1 L CGXII with 20% (*v*/*v*) AQ medium was used as a batch medium, and either 600 mL CGXII concentrate or CGXII concentrate supplemented with 20% (*v*/*v*) AQ were used as feed medium (Figure S5). Both cultures reached the stationary phase after about 32 h with comparable growth rates (0.21 h^−1^ for CGXII concentrate as feed, 0.19 h^−1^ for CGXII concentrate with 20% (*v*/*v*) AQ as feed). With CGXII concentrate as feed, a maximal ∆OD_600 nm_ of 206, and an astaxanthin concentration of 6.1 mg L^−1^ (cellular content of 0.15 mg g^−1^) accumulated during 64 h with a maximum productivity of 0.10 mg L^−1^ h^−1^ (Figure S5A). When AQ was added to the feed, the ∆OD_600 nm_ was 208 and an astaxanthin concentration of 3.8 mg L^−1^ (cellular content of 0.08 mg g^−1^ CDW) accumulated during 64 h with a maximum volumetric productivity of 0.08 mg L^−1^ h^−1^ (Figure S5B). Therefore, the beneficial effect of AQ supplementation is stable at larger scales, at least in the batch phase. However, additional supplementation of AQ via the feed did not further increase production. H_3_PO_4_ was used as acid for pH adjustments in all fermentations. The H_3_PO_4_ consumption during both batch fermentations was comparable, as was the consumption during both fed-batch fermentations. Therefore, the addition of H_3_PO_4_ did not lead to significant differences in the phosphorus source composition of the cultivation media, and all occurring effects can be attributed to the presence or absence of AQ in the medium. The total carotenoid content was more than doubled in both fed-batch fermentations (max. 72.7 mg L^-1^ when fed with CGXII concentrate, and max. 106 mg L^−1^ when fed with CGXII concentrate plus 20% (*v*/*v*) AQ, respectively) compared to the batch fermentations (max. 24.8 mg L^-1^ in CGXII and max. 30.3 mg L^-1^ in CGXII plus 20% (*v*/*v*) AQ) (Figure S6). Thus, the best conditions for astaxanthin production using AQ by *C. glutamicum* strain ASTA* required supplementation of 20% (*v*/*v*) AQ in the batch phase, but not during the feeding phase.

## 3. Discussion

In this study, a promising example with potential for the circular economy was presented. The liquid phase of a sidestream from a recirculating aquaculture system for salmon served as a sustainable feedstock for *C. glutamicum*. Fermentative production of several carotenoids, including the aquaculture feed ingredient astaxanthin was increased by usage of AQ.

Growth of *C. glutamicum* was supported by AQ as the sole medium component. However, it was a poor source of carbon and nitrogen. The addition of glucose, urea, and/or ammonium sulfate restored the growth to levels comparable with the standard medium. While L-glutamine, L-glutamate and a number of other amino acids present in AQ, e.g., L-alanine, L-asparagine, L-serine, and L-threonine, can be used as nitrogen sources by *C. glutamicum* [95], their concentration was comparatively low. Other amines present in AQ, such as putrescine, cadaverine, and 5-AVA, cannot be utilized as nitrogen sources by *C. glutamicum* [96,97,98]. Likely, nitrites and nitrates are present in AQ. *C. glutamicum* is able to metabolize nitrate to nitrite as part of a respiratory chain [99,100]; however, neither is used as a nitrogen source. In RAS, nitrogen is typically removed from the circulating water, e.g., by biofilter arrangements containing microbial communities of nitrifying and denitrifying microorganisms [101,102]. Prior to the demonstration that AQ supported growth and carotenoid production by *C. glutamicum*, it was reported that the supplementation of an aquaculture sidestream with aquaculture sludge improved growth and omega-3 fatty acid production of an algal co-cultivation [21].

Carotenoid production by *C. glutamicum* was improved by supplementation with AQ. However, since AQ is a complex source of macro- and micro-elements, it is very difficult to speculate which component(s) cause the improvement. It is tempting to hypothesize that trace elements may be involved as several enzymes of carotenogenesis use metal ions as cofactors. For example, prenyltransferases use divalent metal ions, such as Mg^2+^ and Mn^2+^ as cofactors [103,104,105]. In *C. glutamicum*, the geranylgeranyl pyrophosphate synthases IdsA and CrtE require Mg^2+^ for their activity [106]; however, Mg and MgO concentrations in AQ were below the detection limit (<3 g L^−1^). It is not known whether the substrate specificities of the prenyltransferases used here (IdsA, CrtE, CrtB, CrtEb, or LbtBC) are affected when Mg^2+^ is replaced by Mn^2+^, as was recently observed for a flavonoid prenyltransferase from *Artocarpus heterophyllus* [107]. Ferredoxin is involved as an electron carrier in several steps of decaprenoxanthin biosynthesis in *C. glutamicum.* For example, 28 reduced ferredoxins are required for the biosynthesis of the isoprenoid diphosphate precursors DMAPP, IPP and HMBPP, while their conversion to decaprenoxanthin yields only three reduced ferredoxins [61]. For the biosynthesis of zeaxanthin and astaxanthin, four additional reduced ferredoxins are required for the reactions catalyzed by CrtZ [108]. These iron-sulfur cluster containing enzymes of carotenoid biosynthesis have a high demand for sulfur and iron to be provided by the medium. In this respect, it is noteworthy that supplementation with AQ (containing 1 g L^−1^ S and 2 g L^−1^ SO_4_) increased sulfur availability. Taken together the diverging effects of AQ supplemented CGXII and CGAQ on the production of the tested native and heterologous carotenoids prompt that the production of each carotenoid may be improved by optimization of the (trace) element composition of the growth medium. Earlier studies in carotenoid producing yeast [109,110] and bacteria [111,112] suggest that the optimum concentration ratios for trace elements have to be evaluated with regards to the production host, the involved enzymes, their cofactors, and further reaction partners.

Bacterial astaxanthin production from AQ was demonstrated here for the first time. Previously, sidestream derived astaxanthin production was described for the yeast *Xanthophyllomyces dendrorhous*. Cultures of *X. dendrorhous* produced up to 9.69 µg L^−1^ astaxanthin when grown for five days on pre-treated whey, a sidestream of the dairy industry (volumetric productivity of 0.08 µg L^−1^ h^−1^), and up to 1.88 mg L^−1^ astaxanthin when cultivated in a fruit and vegetable waste derived medium for 7 days (0.01 mg L^−1^ h^−1^) [113]. Moreover, 26 mg L^−1^ astaxanthin were produced by *X. dendrorhous* after about 200 h of cultivation (0.13 mg L^−1^ h^−1^) in a partially-saccharified mussel wastewater [114]. In a co-cultivation of the bacterium *Bacillus subtilis* and the alga *H. pluvialis* for astaxanthin production from a starch-containing sidestream of a potato processing plant a titer of 8 mg L^−1^ astaxanthin was achieved after 350 h of cultivation (0.02 mg L^−1^ h^−1^) [115]. Our study revealed a much faster astaxanthin production (7.4 mg L^−1^ in 48 h; 0.15 mg L^−1^ h^−1^) upon supplementation with AQ and partially also higher titers than those achieved by *X. dendrorhous*. The yeast *P. rhodozyma* produced about 130 mg L^−1^ astaxanthin after 120 h by direct fermentation of food wastes (1.08 mg L^−1^ h^−1^) [116]. On stillage media derived from bagasse-based ethanol fermentation of *S. cerevisiae*, about 18 mg L^−1^ astaxanthin accumulated within one week of fermentation (0.10 mg L^−1^ h^−1^) [117]. Both sidestreams are much richer as compared to the AQ used in this study. The total carotenoid concentrations of up to about 0.1 g L^−1^ observed in this study may indicate that more astaxanthin can be produced if all precursor carotenoids are converted to astaxanthin. This may be approached by adjusting the copy number and/or expression levels of *crtZ* and *crtW* as previously demonstrated for *E. coli* and *S. cerevisiae* [118,119].

In aquatic feeds, bacterial biomass proved to be a sustainable, protein rich substrate to partially or completely replace traditional protein sources, such as fish meal [120]. In aquaculture feed formulations, phototrophic purple bacteria [121,122], lactic acid bacteria [123], and methanotrophic bacteria [124,125] have been used. Feeding whole cells of *C. glutamicum* to fish or crustaceans may be an option to provide astaxanthin and additional components relevant as feed additives. For this application, the strain ASTALYS* may be beneficial as it secretes L-lysine to the culture medium and accumulates astaxanthin in the cells [126]. Lysine typically is deficient in aquatic feeds [127,128] and astaxanthin is used in flesh and/or shell coloration of several aquaculture reared fish and crustaceans [24,127]. Whole cells obtained by spray-drying fermentation broth of L-lysine producing *C. glutamicum* were already commercialized for poultry feeds [95]. Thus, spray-dried fermentation broth of *C. glutamicum* ASTALYS* may support growth of poultry and coloration of egg yolks. In this regard, it has to be noted that several amino acid producing *C. glutamicum* strains have been approved by the European Food Safety Authority (EFSA) as a feed additive for all animal species [129]. Today, the establishment of a circular economy-based fish farm, including fish tanks and bioreactors, might be visionary. However, on-site production of the fish feed ingredients by cultivation of bacteria and algae on the sidestreams from the fish tanks would not only reduce the energy consumption of RAS due to reduced transport of feed ingredients [7], but would be one step closer towards a sustainable society.

## 4. Materials and Methods

### 4.1. Preprocessing of the Aquaculture Sidestream

The aquaculture sidestream was collected from the sump of a post-smolt RAS for salmon operated by Lumarine AS (Tjeldbergodden, Norway) outside of Trondheim (Norway). A plastic canister was filled with aquaculture sidestream, and was left to settle by gravitation for 0.5 h. The supernatant (liquid aquaculture sidestream) was thereafter gently poured off, frozen (−20 °C), and transported to our research facility in Bielefeld (Germany). The liquid aquaculture sidestream was defrosted, stirred, and frozen (−20 °C) in smaller containers until further use. Preprocessing of the liquid aquaculture sidestream in order to use it as a growth medium component was implemented by a 90 min centrifugation step at 4000 rpm and subsequent sterile filtration of the supernatant. The filtration was performed with a vacuum driven Steritop^®^ (Millipore, Burlington, MA, USA) with 0.22 µm pore diameter. The resulting AQ was used in the growth experiments.

### 4.2. Microorangisms and Cultivation Conditions

Strains and plasmids used in this study and their characteristics and references are listed in Table 1. Chemicals were delivered by Carl Roth (Karlsruhe, Germany), if not stated differently. Precultures were grown in 100 mL or 500 mL shake flasks at 30 °C and 120 rpm, or 37 °C and 180 rpm for *C. glutamicum* or *E. coli* respectively. Precultures of *E. coli* DH5α, *C. glutamicum* ATCC 13032, and derived strains were grown in LB medium [130]. *C. glutamicum* ASTALYS* precultures were grown in BHIS (37 g L^−1^ BHI, 91 g L^−1^ Sorbitol) supplemented with 10 g L^−1^ glucose. For main cultures CGXII minimal medium (20 g L^−1^ (NH_4_)_2_SO_4_, 1 g L^−1^ K_2_HPO_4_, 1 g L^−1^ KH_2_PO_4_, 5 g L^−1^ urea, 42 g L^−1^ MOPS buffer, 0.2 mg L^−1^ biotin, 30 mg L^−1^ PCA, 10 mg L^−1^ CaCl_2_, 250 mg L^−1^ MgSO_4_·7 H_2_O, trace elements: 10 mg L^−1^ FeSO_4_·7 H_2_O, 10 mg L^−1^ MnSO_4_·H_2_O, 0.02 mg L^−1^ NiCl_2_·6 H_2_O, 0.313 mg L^−1^ CuSO_4_·5 H_2_O, and 1 mg L^−1^ ZnSO_4_·7 H_2_O) [95] supplemented with 40 g L^−1^ glucose was used as a control. Comparisons of AQ-based or -supplemented media were carried out using 20% (*v*/*v*) AQ. When appropriate, 25 µg mL^−1^ kanamycin, 100 µg mL^−1^ spectinomycin, and 1 mM IPTG were added to the medium. Cultures were inoculated to an initial OD_600 nm_ of 1 after washing in TN buffer (50 mM Tris, 50 mM NaCl and pH 6.3). For OD measurements a Shimadzu UV-1202 spectrophotometer (Duisburg, Germany) was used. Growth experiments were conducted in 1 mL scale with gas permeable sealing foil in a flowerplate of the Biolector^®^ microcultivation system (m2p-labs GmbH, Baesweiler, Germany) at 30 °C and 1100 rpm in triplicates.

### 4.3. Fermentative Production

A glass bioreactor with a total volume of 3.7 L (KLF, Bioengineering AG, Switzerland) was used for the fermentations. Two six-bladed Rushton turbines were placed on the stirrer axis with a distance of 6 cm and 12 cm from the bottom of the reactor. A mechanical foam breaker was installed on the stirrer axis with a distance of 22 cm to the bottom of the reactor. The stirrer to reactor diameter ratio was 0.39, and the aspect ratio of the reactor was 2.6:1.0. A steady airflow of 1 NL min^−1^ was maintained from the bottom through a ring-sparger. The fermentations were performed with a head space overpressure of 0.3 bar. An automatic control of the stirrer speed between 400 rpm and 1500 rpm kept the relative dissolved oxygen saturation (rDOS) at 30%. The temperature was kept at 30 °C, and a pH of 7.0 was automatically maintained by the addition of 10% (*v*/*v*) H_3_PO_4_ and 4 M KOH. For inoculation a first pre-culture was grown in 10 mL LB medium with addition of 10 g L^−1^ glucose and 25 µg mL^−1^ kanamycin in a 100 mL shake flask. A second pre-culture in 200 mL CGXII medium with addition of 40 g L^−1^ glucose and 25 µg mL^−1^ kanamycin in a 2 L shake flask was inoculated to an OD_600 nm_ of 1. For the batch fermentation an initial working volume of 2 L was inoculated to an OD_600 nm_ of 2 from the second pre-culture. The fed-batch fermentations were performed with an initial working volume of 1 L, inoculated to an OD_600 nm_ of 2 with the second pre-culture. An amount of 600 mL feed medium (CGXII concentrate) was used with the following formulation: 433.7 g L^−1^ glucose, 34.4 g L^−1^ (NH_4_)_2_SO_4_, 8.7 g L^−1^ K_2_HPO_4_, 8.7 g L^−1^ KH_2_PO_4_, 8.6 g L^−1^ Urea, 5.2 g L^−1^ MgSO_4_·7 H_2_O, 100 mg L^−1^ FeSO_4_·7 H_2_O, 100 mg L^−1^ MnSO_4_·H_2_O, 0.2 mg L^−1^ NiCl_2_·6 H_2_O, 3.13 mg L^−1^ CuSO_4_·5 H_2_O, 10 mg L^−1^ ZnSO_4_·7 H_2_O, 1 mg L^−1^ Biotin, and 6 mL L^−1^ Antifoam 204. If indicated, 20% (*v*/*v*) AQ was added to this feed formulation. A steady airflow of 0.5 NL min^−1^ was maintained from the bottom through a ring sparger for the first 24 h of cultivation, afterwards the airflow was increased to 1 NL min^−1^. The feed was primed when the rDOS fell below 30% for the first time. The feed pump activated every time the rDOS exceeded 60% and stopped when it subsequently fell below 60%, to prevent oversaturation with glucose. Foam formation during the fermentation was reduced by addition of antifoam 204 controlled via an antifoam probe. Samples during the fermentations were collected with an autosampler and cooled down to 4 °C until further use.

### 4.4. Recombinant DNA Work

The pSH2 expression vector was constructed by site-directed mutagenesis of the *repA* gene in the pSH1 vector. This mutation (exchange of Gly at position 429 of RepA protein by Glu) resulted in an increased plasmid copy number. Site-directed mutagenesis was performed via plasmid backbone amplification (primer HA36 + HA37; see Table 2) with *Pfu* Turbo DNA Polymerase (Agilent, Santa Clara, CA, USA). The plasmids pSH2_*crtZ_Fp_* and pSH2_*crtW_Fp_* were constructed in *E. coli* DH5α on the basis of pSH2. Polymerase chain reaction (PCR) fragments of *crtZ_Fp_* and *crtW_Fp_* were generated from *Fulvimarina pelagi* using the primers HA34 + HA35, and FpW1 + FpW4, respectively. The PCR products were cloned into the BamHI (Thermo Fisher Scientific, Waltham, MA, USA) digested pSH2 vector by Gibson assembly [136]. The CaCl_2_-competent *E. coli* DH5α were prepared and transformed via heat shock [137]. Transformants were screened by colony PCR using the primers PD5 and 582. Plasmid isolates were prepared with a plasmid miniprep kit (GeneJet, Thermo Fisher Scientific, Schwerte, Germany), and confirmed via sequencing with primers PD5 and 582. *C. glutamicum* strains were transformed by electroporation [138]. For the construction of ASTALYS*, BETALYS [126] was transformed with pSH1_*crtZ*~*W* [46].

### 4.5. Quantification of Macro- and Micronutrients

A 1·10^−2^ dilution of the untreated liquid aquaculture sidestream in water was sent to Eurofins Agraranalytik Deutschland GmbH (Jena, Germany) to analyze the pH, organic substance content, and total nitrogen, ammoniacal nitrogen, phosphorous, phosphorous as P_2_O_5_, potassium, potassium as K_2_O, magnesia, magnesia as MgO, calcium, calcium as CaO, sulfur, sulfur as SO_4_, boron, manganese, molybdenum, copper, and zinc content of the untreated liquid aquaculture sidestream.

### 4.6. High-Performance Liquid Chromatography (HPLC) Analysis

For all HPLC analysis an Agilent 1200 series system (Agilent Technologies Deutschland GmbH, Böblingen, Germany) was used. Culture samples (200 or 500 µL) were centrifuged at 14,000 rpm for 10 min, the supernatants and pellets were stored separately at −20 °C until analysis.

#### 4.6.1. Quantification of Amino Acids and Amines

For the analysis of extracellular amino acids and their derivatives 5 µL sample and an automatic pre-column derivatization with *ortho*-phthaldialdehyde (OPA) were used with a reversed-phase main column (LiChrospher 100 RP18 EC-5, 125 × 4.6 mm; CS-Chromatographie Service GmbH, Langerwehe, Germany). l-asparagine was used as an internal standard. The separation was achieved at 40 °C with 0.25% (*v*/*v*) sodium acetate (pH 6.0) (A) and methanol (B) as mobile phases, with the following gradient and flow profile: 0 min 20% B 0.7 mL min^−1^, 3 min 38% B 0.7 mL min^−1^, 6 min 42% B 0.1 mL min^−1^, 7 min 46% B 0.7 mL min^−1^, 14.5 min 70% B 1.2 mL min^−1^, 14.8 min 75% B 1.2 mL min^−1^, 16.8 min 85% B 1.2 mL min^−1^, 17.8 min 20% B 1.2 mL min^−1^, and 19.5 min 20% B 1.2 mL min^−1^ adapted from [139]. The fluorescent derivatives were detected using a fluorescence detector with an excitation wavelength of 230 nm, and an emission wavelength of 450 nm.

#### 4.6.2. Quantification of Carbohydrates and Organic Acids

The carbohydrates in the cultivation medium were quantified with an organic acid resin column (Aminex, 300 mm × 8 mm, 10 µm particle size, 25 Å pore diameter; CS-Chromatographie Service GmbH, Langerwehe, Germany) under isocratic conditions with a flow of 0.8 mL min^−1^ as described previously [80]. The analytes were detected using a refractive index detector (RID) and a diode array detector (DAD) at 210 nm.

#### 4.6.3. Quantification of Carotenoids

The analysis of carotenoids was performed as previously described [61]. Samples were extracted until the remaining pellet of cell debris was colorless; no further analysis in regards to recovery percentage and purity of the extracted carotenoids was performed. Carotenoid analysis was performed for all growth and production experiments in this study. For quantification of the carotenoid contents the peaks detected at 471 nm were used. Decaprenoxanthin, BABR, C.p. 450, and sarcinaxanthin contents presented in the results section were standardized with β-carotene.

## 5. Conclusions

The preprocessed liquid phase of a sidestream from a recirculating aquaculture system for salmon was shown to be suitable to support growth and carotenoid production by *C. glutamicum*. The beneficial effect of adding this sidestream to growth media was observed for strains overproducing either native or non-native carotenoids. In particular, astaxanthin production more than doubled upon AQ supplementation in small-scale cultivation, and in 2 L batch and fed-batch bioreactor fermentations. Thus, our proof-of-principle example for production of the fish feed supplement astaxanthin from AQ holds the potential to contribute to the establishment of the circular economy in aquaculture.

## Figures and Tables

**Table 1:** Strains and plasmids used in this study.

Strain/Plasmid	Characteristics	Reference
*Escherichia coli* DH5α	F^-^ *thi*^−1^ *endA1 hsdR17*(r^-^, m^-^) *supE44* ∆*lacU169* (Φ80*lacZ*∆M15) *recA1 gyrA96*	[131]
*Fulvimarina pelagi*	Type strain, HTCC 2506; DSM No. 15513	[132]
***Corynebacterium glutamicum* strains**		
WT	wild type, ATCC 13032	[133]
MB001	ATCC 13032 with in-frame deletion of prophages cgp1 (cg1507-cg1524), cgp2 (cg1746-cg1752), cgp3 (cg1890-cg2071)	[134]
MB001∆*crtR*	MB001 derivative with deletion of *crtR* (cg0725)	[57]
LYC5	MB001 derivative with deletion of *crtY_ef_Eb* (cg0717-cg0719), and chromosomal integration of P*tuf*-*dxs* and P*tuf*-*crtEBI*	[58]
LYC6	LYC5 derivative with deletion of *crtR* (cg0725)	[57]
BABR1	LYC5 carrying pVWEx1_*lbtBC* and pEKEx3_*sigA*	[56]
CP1	LYC6 carrying pEKEx3_*lbtABC*	[57]
SAX1	LYC6 carrying pEKEx3_*crtE2Y*	[57]
BETA4	LYC6 with chromosomal integration of *crtY* from *P. ananatis* under the control of *tuf* promotor	[57]
ZEA5	BETA4 carrying pSH2_*crtZ_Fp_*	this work
CAN5	BETA4 carrying pSH2_*crtW_Fp_*	this work
ASTA*	BETA4 carrying pSH1_*crtZ*~*W_Fp_*	[46]
BETALYS	GRLys1 with the following modifications: ∆*ldhA* (cg3219), ∆*sugR* (cg2115), ∆*crtR* (cg0725), ∆*crtY_ef_Eb* (cg0717-cg0719), chromosomal integration of P*tuf*-*crtEBI* and P*tuf*-*crtY_Pa_*	[126]
ASTALYS*	BETALYS carrying pSH1_*crtZ*~*W_Fp_*	this work
**Plasmids**		
pEKEx3_*sigA*	Spec^R^; pBL1 *oriV_Cg_*, *E. coli*/*C. glutamicum* shuttle vector; for IPTG-inducible expression of *sigA* from *C. glutamicum*	[135]
pEKEx3_*crtE2Y*	Spec^R^; pBL1 *oriV_Cg_*, *E. coli*/*C. glutamicum* shuttle vector; for IPTG-inducible expression of *crtE2* and *crtYg*/*h* from *M. luteus* containing an artificial ribosome binding site in front of *crtE2*	[48]
pEKEx3_*lbtABC*	Spec^R^; pBL1 *oriV_Cg_*, *E. coli*/*C. glutamicum* shuttle vector for IPTG-inducible expression of codon optimized *lbtABC* from *Dietzia sp.* CQ4 containing artificial ribosome binding sites in front of each gene	[48]
pVWEx1_*lbtBC*	Km^R^; pCG1 *oriV_Cg_*, *E. coli*/*C. glutamicum* shuttle vector for IPTG-inducible expression of *lbtBC* from *Dietzia* sp. CQ4	[56]
pSH1_*crtZ*~*W_Fp_*	Km^R^; pHM519 oriV*_Cg_*; *E. coli*/*C. glutamicum* shuttle vector, P*tuf*, encoding a fusion protein comprising CrtZ and CrtW from *F. pelagi*	[46]
pSH2	pSH1 derivative with mutation in *repA*	this work
pSH2_*crtZ_Fp_*	pSH2 derivative for constitutive expression of *crtZ* from *F. pelagi*	this work
pSH2_*crtW_Fp_*	pSH2 derivative for constitutive expression of *crtW* from *F. pelagi*	this work

**Table 2:** Oligonucleotides used in this study.

Oligonucleotide	Sequence (5′→3′)
HA36	AAAATCGCTTGACCATTGCAGGTTG
HA37	CTTTAGCTTTCCTAGCTTGTCGTTGAC
HA34	CATGCCTGCAGGTCGACTCTAGAGGAAAGGAGGCCCTTCAGATGACGATCTGGACTCTCTACTAC
HA35	ATTCGAGCTCGGTACCCGGGGATCTTACCGAACCGGCGCGT
FpW1	CATGCCTGCAGGTCGACTCTAGAGGAAAGGAGGCCCTTCAGATGACCCTCAGCCCAACCTC
FpW4	ATTCGAGCTCGGTACCCGGGGATCTTAGGACTGGCGAGTATGCG
PD5	CGCTCACCGGCTCCAGATTTATCAG
582	ATCTTCTCTCATCCGCCA

## Data Availability

Not applicable.

## Supplementary Materials

The following supporting information can be downloaded at: https://www.mdpi.com/article/10.3390/molecules28041996/s1, Table S1: Amino acid concentrations in the aquaculture sidestream, Figure S1: Chromatogram of the amino acid analysis of the aquaculture sidestream, Figure S2: Chromatogram of the carbohydrate analysis of the aquaculture sidestream, Figure S3: Carotenoid content of *C. glutamicum* strains grown in AQ supplemented media, Figure S4: Growth of *C. glutamicum* strains in AQ supplemented media, Figure S5: Fed-batch fermentations, Figure S6: Carotenoid content of *C. glutamicum* ASTA* during batch and fed-batch fermentations.
