# Supplementary material for: From Aquaculture to Aquaculture: Production of the Fish Feed Additive Astaxanthin by Corynebacterium glutamicum Using Aquaculture Sidestream
Source: Molecules. 2023 Feb 20;28(4):1996. doi: 10.3390/molecules28041996 (PMC9958746; doi:10.3390/molecules28041996)
Supplement: Supplementary file 1 [file molecules-28-01996-s001.zip › molecules-2134463-supplementary.pdf]

Supplementary Material

Table S1: Amino acid concentrations in the aquacultrue sidestream. The peaks of Glycine and Threonine, as well as Tyrosine, Arginine, Alanine and GABA co-eluted and could not be detected separately (see Figure S1), therefore the given data for these components are not reliable and indicated in grey.

Amino acid / Diamine	Concentration [mg/L]
L-aspartatic acid	10.7
L-glutamate	8.1
L-serine	3.8
L-glutamine	0.9
L-histidine	0.7
L-citrulline	6.2
L-glycine	4.8
L-threonine	1.6
L-tyrosine	6.3
L-arginine	55.2
L-alanine	2.4
GABA	1046.5
5AVA	76.0
L-tryptophane	0.5
L-methionine	14.1
L-valine	9.9
L-phenylalanine	17.9
L-isoleucine	10.8
L-leucine	4.6
L-ornithine	0.7
L-lysine	0.5
L-putrescine	61.5
Cadaverine	52.9

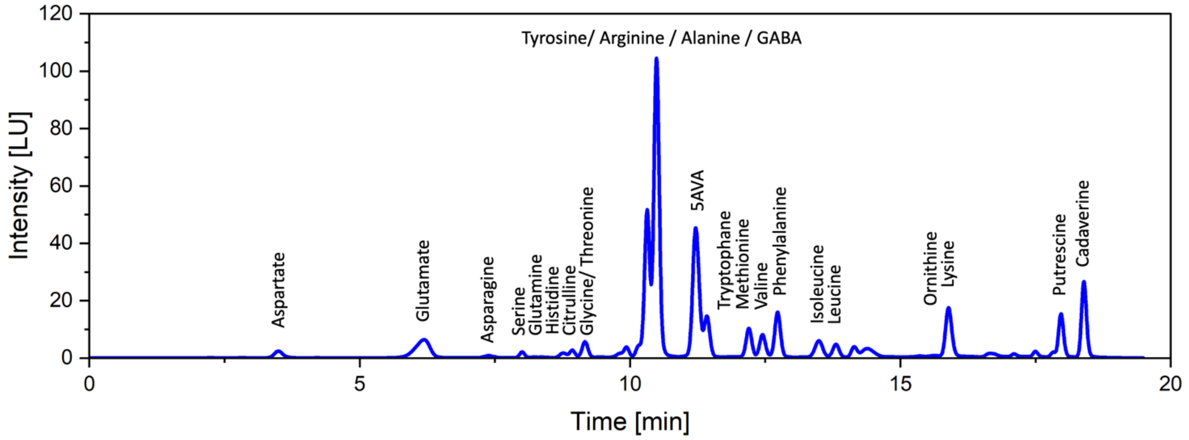

Figure S1: Chromatogram of the amino acid analysis of the aquaculture sidestream.

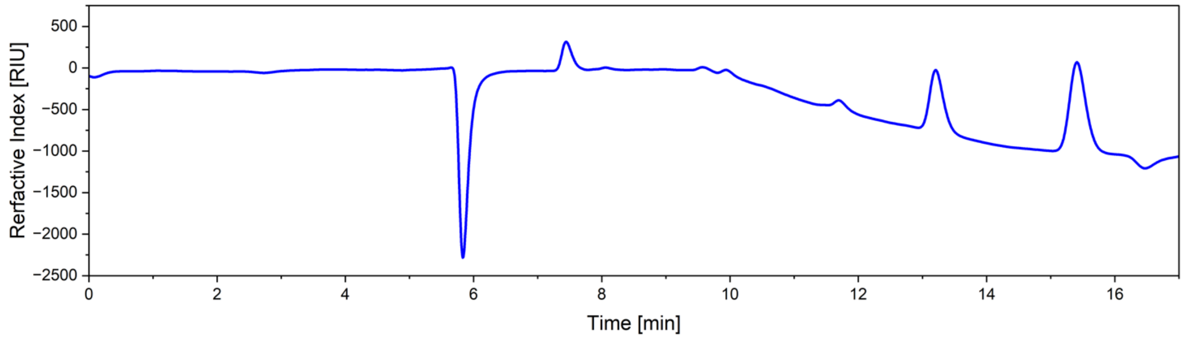

Figure S2: Chromatogram of the carbohydrate analysis of the aquaculture sidestream.

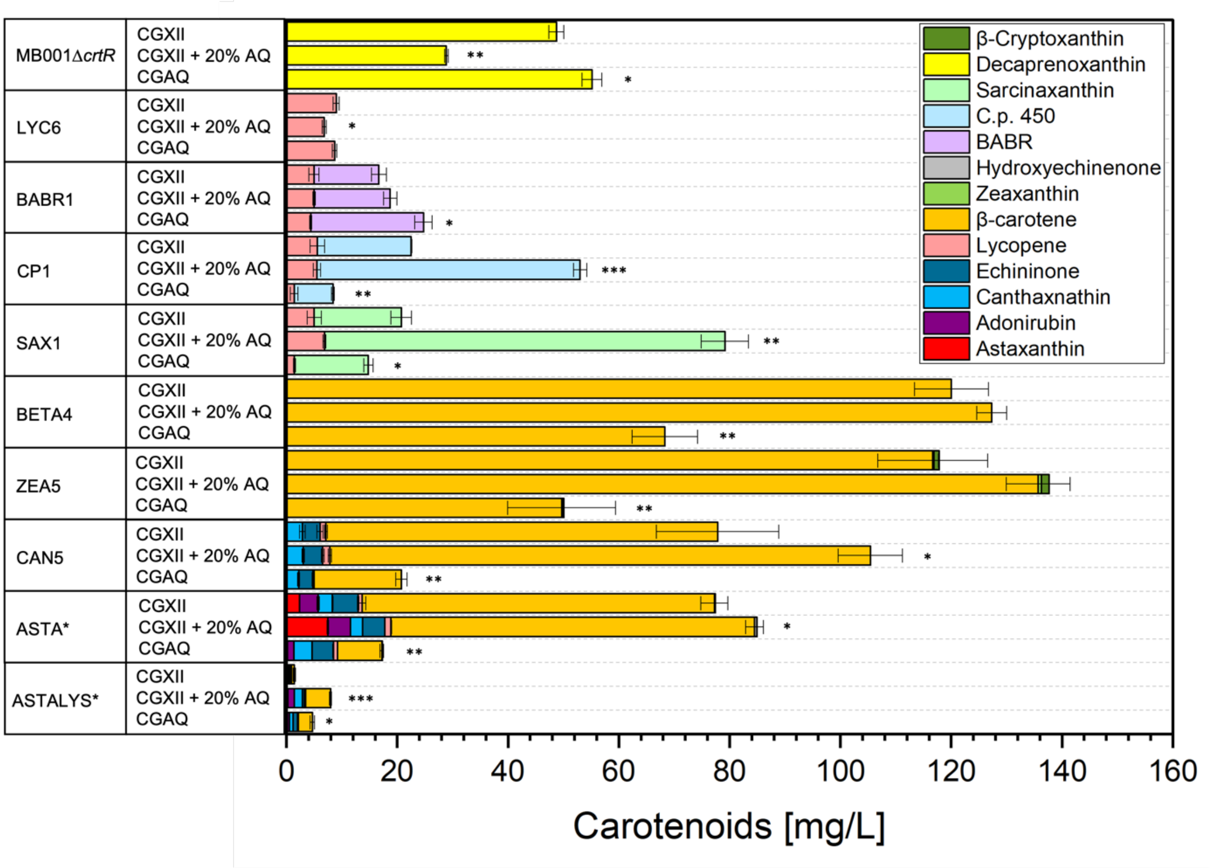

Figure S3: Carotenoid content of *C. glutamicum* strains grown in AQ supplemented media. Carotenoid contents of *C. glutamicum* MB001 Δ*crtR*, LYC6, CP1, BABR1, SAX1, BETA4, ZEA5, CAN5, ASTA* and ASTALYS* grown on CGXII, CGXII supplemented with 20 % (v/v) of the aquaculture sidestream (AQ), or the aquaculture derived medium CGAQ for 48 h. Values and error bars represent means and standard deviations of triplicate cultivations. Statistical significance of total carotenoid content in comparison to the cultivation of each strain in CGXII medium was assessed in Student´s t-test (*** $\rho < 0.0001$, ** $\rho < 0.001$, * $\rho < 0.05$).

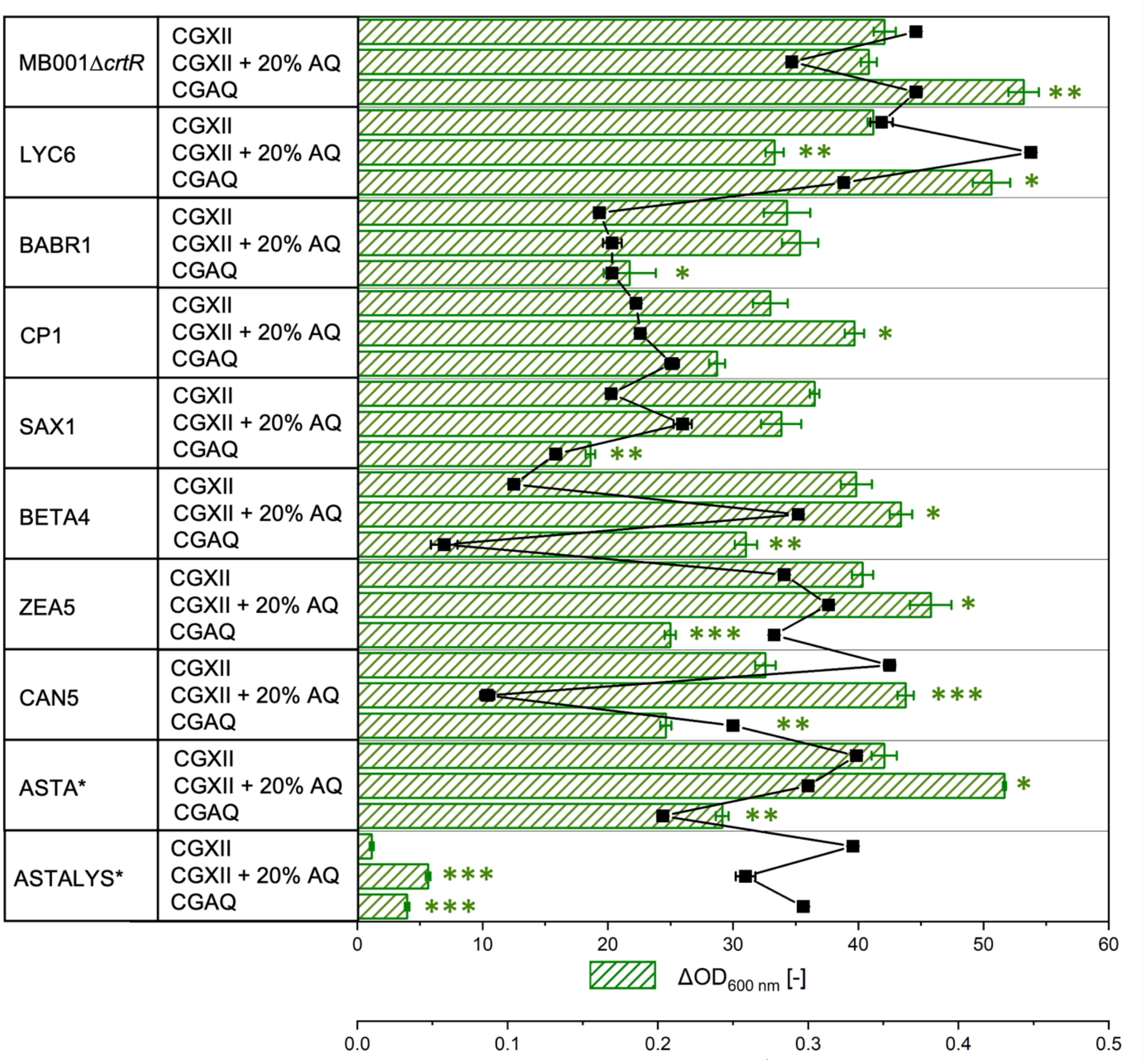

Figure S4: Growth of *C. glutamicum* strains in AQ supplemented media. $\Delta OD_{600\ nm}$ (bars), μ_{max} (black squares) of *C. glutamicum* MB001 Δ*crtR*, LYC6, CP1, BABR1, SAX1, BETA4, ZEA5, CAN5, ASTA* and ASTALYS* grown on CGXII, CGXII supplemented with 20 % (v/v) of the aquaculture sidestream (AQ), or the aquaculture derived medium CGAQ. Values and error bars represent means and standard deviations of triplicate cultivations. Statistical significance of $\Delta OD_{600\ nm}$ in comparison to the cultivation of each strain in CGXII medium was assessed in Student´s t-test (***$\rho < 0.0001$, **$\rho < 0.001$, *$\rho < 0.05$).

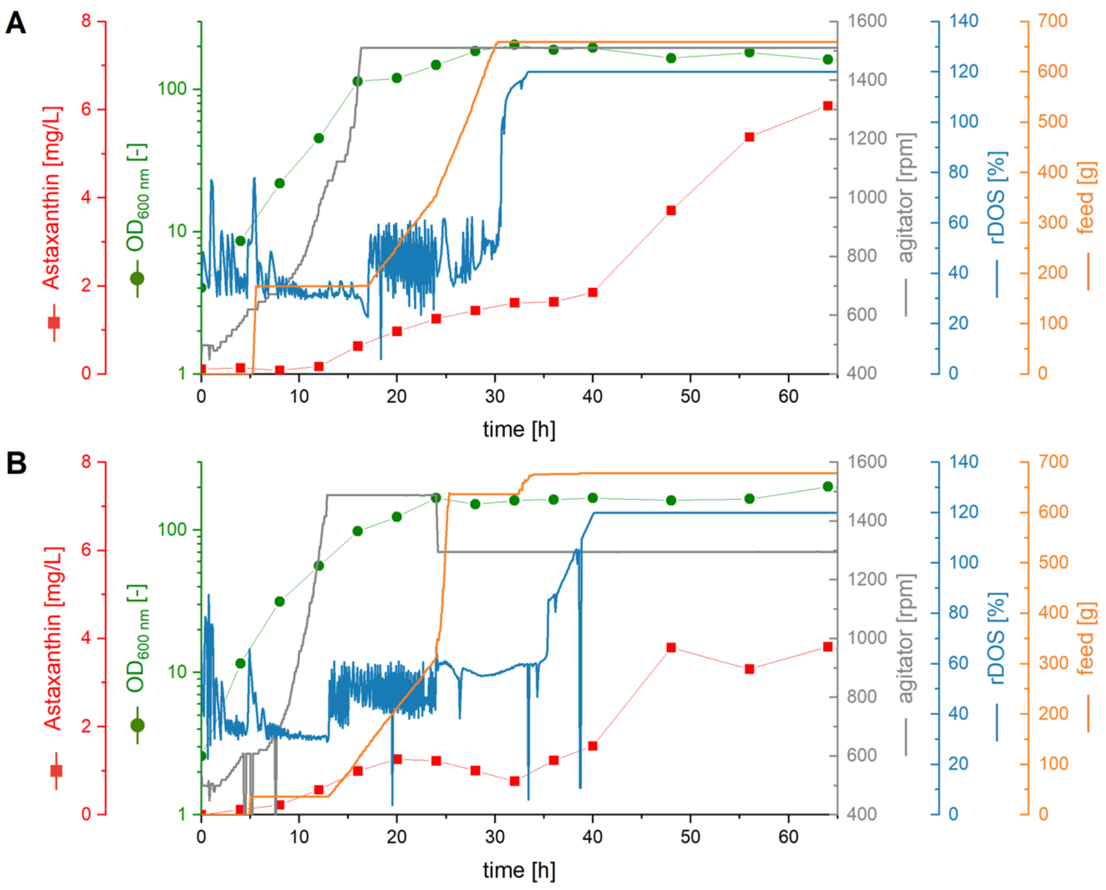

Figure S5: Fed-batch fermentations. Progression of astaxanthin content (red squares), $OD_{600\ nm}$ (green dots), agitator speed (grey line), relative dissolved oxygen concentration (rDOS) (blue line) and feed addition over time (orange line), during fed-batch fermentations with *C. glutamicum* ASTA*. The batch medium for both fermentations was 1 L CGXII with the addition of 20 % AQ, 0.6 L CGXII-concentrate (A) or CGXII-concentrate + 20% AQ (B) were used as feed.

The observed oscillations in rDOS at the beginning of the fermentations were due to an overregulation of the stirrer. This effect is a disadvantage of the used proportional integral derivative (PID) controllers, which is often observed due to the high sensitivity of the rDOS probes towards fluctuations in the dissolved oxygen concentration. Furthermore, the feed was added in a rDOS dependent matter, which led to changes in rDOS every time feed was added to the fermenters.

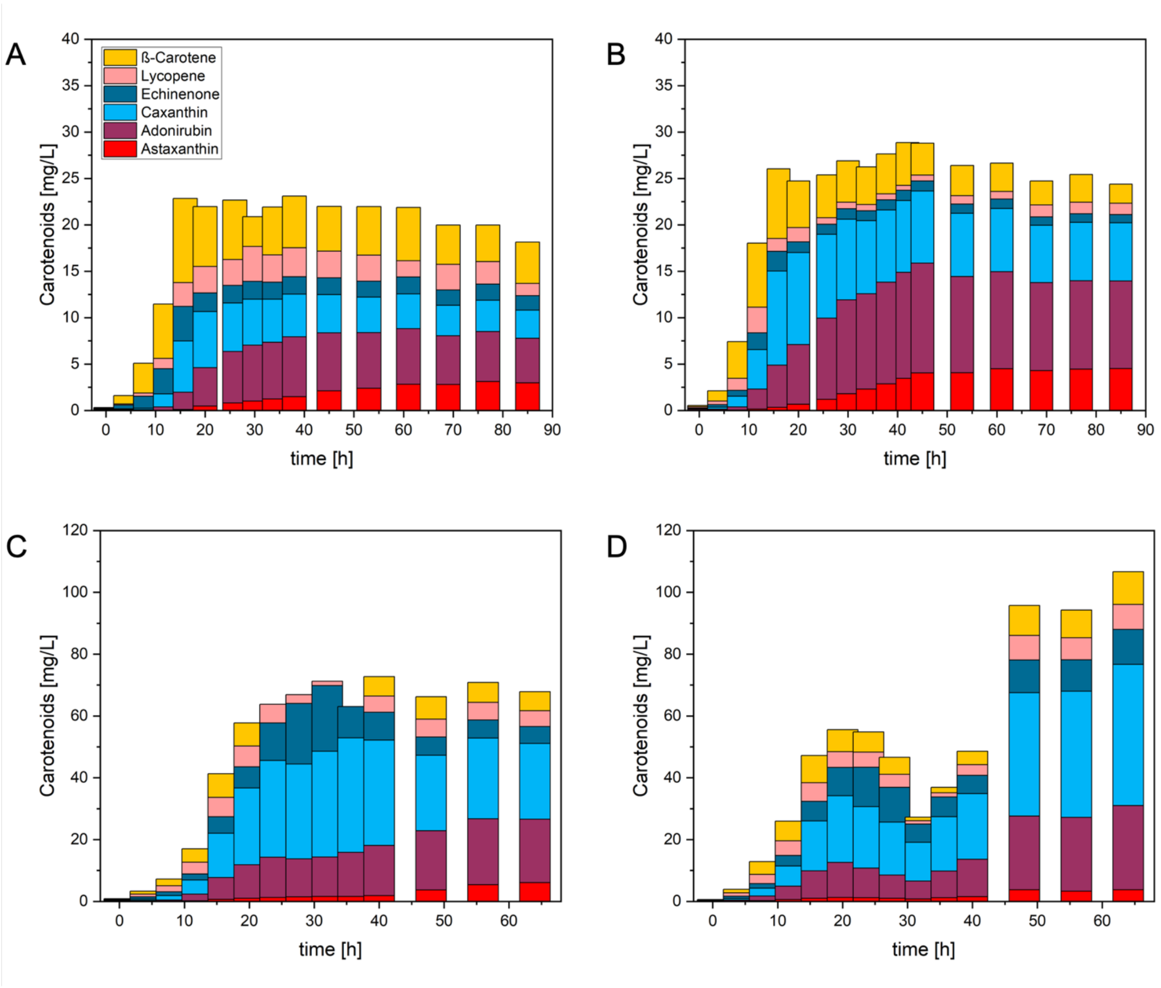

Figure S6: Carotenoid content of *C. glutamicum* ASTA* during batch and fed-batch fermentations. Carotenoid content during 2 L batch fermentations in CGXII medium (**A**) or CGXII supplemented with 20 % (v/v) AQ (**B**) and fed-batch fermentations in 1 L CGXII + 20 % (v/v) AQ fed with either 600 ml CGXII concentrate (**C**) or 600 ml CGXII concentrate supplemented with 20 % (v/v) AQ (**D**).
